# A forgotten diagnosis in right heart failure: A case report and literature review

**DOI:** 10.1002/ccr3.3938

**Published:** 2021-02-23

**Authors:** Azin Alizadehasl, Bahar Galeshi, Mehdi Peighambari, Hamidreza Pouraliakbar, Maryam Moradian, Arash Hashemi

**Affiliations:** ^1^ Cardio‐Oncology Research Center Rajaie Cardiovascular Medical and Research Center Iran University of Medical Sciences Tehran Iran; ^2^ Rajaie Cardiovascular Medical and Research Center Iran University of Medical Sciences Tehran Iran; ^3^ Heart Valve Disease Research Center Rajaie Cardiovascular Medical and Research Center Iran University of Medical Sciences Tehran Iran; ^4^ Erfan General Hospital Tehran Iran

**Keywords:** carcinoid heart involvement, carcinoid syndrome, heart valve surgery, neuroendocrine tumor, right heart failure, tricuspid regurgitation

## Abstract

Carcinoid heart disease is a well‐known complication of carcinoid syndrome that affects morbidity and mortality. Carcinoid heart disease may be asymptomatic in the early stages; therefore, patients with carcinoid syndrome should be screened to prevent misdiagnosis.

## Graphical abstract

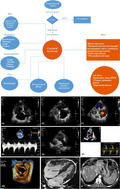

## 1 INTRODUCTION

Neuroendocrine neoplasms, albeit rare, can beget carcinoid tumors and carcinoid heart disease, with the latter considered a prominent etiology in the field of intrinsic right heart valve disorders, leading to right heart failure and considerable morbidity and mortality. So clinicians need to pay heed to its appropriate diagnosis and prevention.

Neuroendocrine neoplasia constitutes infrequent neoplasms that mostly originate from the gastrointestinal tract. They are reported in 2.5‐5 persons per 100 000 people.^1^ The secretion of vasoactive substances by these tumors is responsible for carcinoid syndrome.^2^ Carcinoid tumors usually develop gradually, over years, and are likely to show few or no symptoms until they are bulky enough to be symptomatic or have metastasized, mostly to the liver, followed by the skeletal and pulmonary systems.^1^, ^3^

One of the common complications of carcinoid syndrome is cardiac involvement due to the direct action of vasoactive substances.^2^ Carcinoid syndrome can occur in up to 60% of cases during the course of the disease, but carcinoid heart disease (CaHD) can be the first manifestation in approximately 20% of patients.^3^, ^4^

As cardiac manifestations are associated with a poor long‐term medical prospect and mortality,^3^, ^5^ the detection of cardiac disease in its early stages has a significant value. Cardiac surgeries such as valve replacement, if performed at the optimal time, significantly contribute to the treatment of symptomatic patients and the improvement of their quality of life.^6^

Herein, we describe a patient with CaHD and review the salient practical aspects of carcinoid syndrome and cardiac involvement and introduce a real case referred to our clinic.

## 2 CASE PRESENTATION

A 75‐year‐old woman was referred for the first time to our cardio‐oncology clinic with the chief complaints of progressive dyspnea, fatigue, peripheral edema, and intermittent palpitation. The patient's medical records, which we reviewed after receiving her informed consent, revealed a previous diagnosis of gastrointestinal neuroendocrine neoplasia (midgut carcinoids) with liver metastasis based on ultrasound imaging and endoscopy 4 years earlier. Since surgery was not suitable for her, treatment was done mostly with the aid of alkalizing agents. With a diagnosis of heart failure, she was also placed on cardiac medications, including aspirin, atorvastatin, losartan, MetoHEXAL, and furosemide, but without regular follow‐ups. Also, remarkable in her past medical history was long‐term hypertension. On presentation to our clinic, a physical examination showed a heart rate of around 80 beats/min, an arterial blood pressure of about 140/75 mm Hg, diminished breath sounds over the right lower lung field, and a normal jugular venous pressure. Cardiac auscultation revealed normal first and second heart sounds and a grade II/VI systolic ejection murmur along the left sternal border. Additionally, lower limb edema was apparent without cyanosis.

The patient had undergone 2D transthoracic echocardiography (TTE) in the past, but its results were not available; consequently, the first step in our clinic saw her undergo a comprehensive 2D TTE examination. The findings were normal left ventricular size with mild systolic dysfunction and diastolic dysfunction; severe right atrial (RA) and right ventricular (RV) enlargement with RV dysfunction; a thickened, retracted, and semi‐mobile tricuspid valve; severe free tricuspid regurgitation caused by the absence of leaflet cooptation; and no valvular stenosis. The assessment of the pulmonary valve also showed thickened, malcoapted valves with severe insufficiency, but without stenosis. Moreover, the left heart valves were mildly thickened, most probably in consequence of aging.

The findings led to a diagnosis of CaHD for the patient. Her dyspnea was mostly related to hypertensive cardiomyopathy, diastolic dysfunction, anemia, and low cardiac output due to RV failure. Her other symptoms could be explained by right heart failure. For the assessment of palpitation, 48‐hour Holter monitoring was performed, which revealed frequent premature atrial and ventricular contractions.

The patient's electrolyte disturbances were corrected through biochemical tests. Further, the medications were adjusted taking into account her tolerance under close monitoring in serial follow‐ups via physical examinations concerning symptoms, the severity of the lower leg edema, blood pressure, heart rate, and laboratory data. The serial follow‐ups were done initially every other week for 1 month for medication adjustments. Thereafter, she was visited 3 months later for symptom evaluation, laboratory tests, and TTE, all of which demonstrated clinical and paraclinical improvements in the signs and symptoms of heart failure. Three months afterward, the patient referred to our clinic for the final follow‐up, which once again confirmed the gradual improvement in her symptoms.

Our case is an instance of a patient whose cardiac symptoms were diagnosed as merely heart failure and, thus, mismanaged. Her overall condition and disease severity rendered her unsuitable for surgery.

## 3 DISCUSSION

**FIGURE 1:**
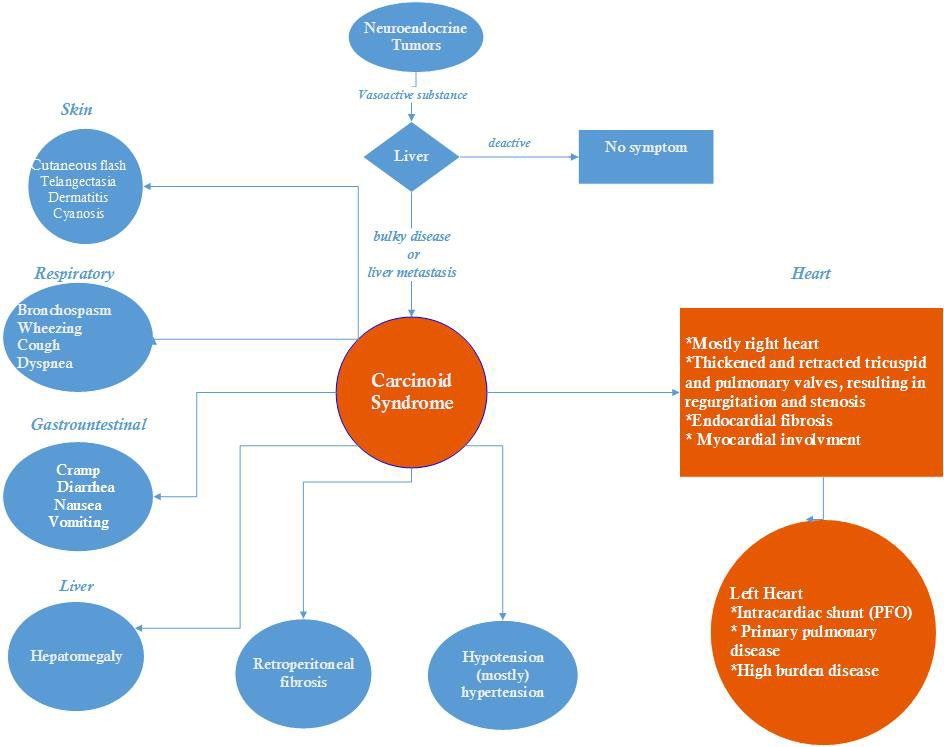
Carcinoid syndrome and its complications

Carcinoid disease is a paraneoplastic syndrome in consequence of the secretion of vasoactive hormones by carcinoid tumors (Figure 1).^2^ Carcinoid tumors could be discovered anywhere in the body, but a digestive and then pulmonary origin is more common.^1^ Chiefly, the tumors arise from the small intestine, commonly in the ileum, and release various kinds of vasoactive chemicals such as serotonin, 5‐hydroxytryptamine (5‐HT), 5‐hydroxytryptophan (5‐HTP), histamine, tachykinins, bradykinins, and prostaglandins into the circulatory system.^2^

Carcinoid tumors usually develop gradually, over years. They tend to exhibit few or no symptoms until they are large enough or have metastasized. The metastasis is predominantly to the liver, where the chemicals secreted by the tumors are usually deactivated; otherwise, they reach the systemic circulation and give rise to the clinical manifestations of carcinoid syndrome.^1^, ^3^ Indeed, between 30% and 40% of patients with carcinoid tumors present with features of carcinoid syndrome.^2^ Nonetheless, about 5% of patients, mainly those with primary ovarian or pulmonary tumors or some cases of midgut tumors with retroperitoneal metastases, might show carcinoid symptoms in the absence of liver metastases.^1^, ^7^ Once large volumes of vasoactive substances reach the right side of the heart, CaHD will appear. Generally, the left side of the heart is spared because these substances are metabolized by the lungs.^2^, ^3^ First described in 1954,^7^ CaHD is expected in more than 50% of affected patients^1^, ^8^ or even up to 70% of cases^7^ in different stages of its severity.^1^, ^8^ A small male predominance (60%) has been described, and a mean age of 56‐63 years at the time of diagnosis has been reported.^8^ Cardiac disease may be detected as an initial presentation of carcinoid syndrome, a well‐known complication that decreases patient survival and causes major morbidity and mortality without therapeutic options.^2^, ^8^, ^9^ Recent decades have, however, witnessed an increase in the survival of patients with CaHD, probably thanks to advances in diagnostic imaging technologies, therapies, cardiac surgeries, and perioperative care. ^1^

The pathogenesis of CaHD is complex and has yet to be elucidated; nevertheless, the 5‐HT receptors are known to play a fundamental role. These receptors, predominantly the 5‐HT2B subtype, are mostly manifested on cardiac valves. The activation of the 5‐HT receptors can trigger the mitogenesis of fibroblasts and smooth muscle cells, the release of inflammatory cytokines, and the upregulation of the transforming growth factor beta‐1. All these changes lead to the deposition of plaque‐like substances on the endocardial surfaces of the valve leaflets, the subvalvular apparatus (chordae and papillary muscles), the chambers, and occasionally the intima of the pulmonary arteries, the aorta, and the venae cavae.^1^ Plaque formation, in turn, sets in motion annular restriction and leaflet thickening, with the resultant significant degenerative changes in the valvular apparatus begetting the severe retraction and non‐coaptation of the valve leaflets.^10^ These structural and valvular lesions ultimately bring about right‐sided heart failure. Notably, what differentiates CaHD from the other etiologies of heart failure is a history of flushing, diarrhea, and pulmonary symptoms.^10^, ^11^ Besides valvular involvement, coronary artery vasospasm is also associated with CaHD and usually presents in patients with a history of nonocclusive coronary artery disease.^3^ In the presence of ischemic heart disease, CaHD can lead to coronary artery vasospasm, angina, and in‐stent thrombosis.^4^ Arrhythmias, albeit infrequent, might be secondary to increased sympathetic hyperactivity caused by vasoactive chemicals.^3^ Atrial and ventricular arrhythmias such as atrial fibrillation, ventricular tachycardia, ventricular fibrillation, and cardiac arrest have also been reported.^4^ Infrequently, in about 4% of patients,^1^ an isolated intracardiac mass may be a manifestation of CaHD without any valvular involvement.^1^, ^4^ These well‐defined, homogeneous, and non‐infiltrative masses are mostly found on either ventricle (including the ventricular septum) during echocardiography.

Carcinoid metastasis to cardiac structures can either be asymptomatic and present as a solitary mass or cause ventricular outflow tract obstruction.^3^ Heart failure owing to pericardial disease and constrictive pericarditis is a rare manifestation of the disease that may present without an obvious valvular disease.^4^

### 3.1 Clinical features of patients with CaHD

The clinical manifestations of CaHD are often unremarkable in the early stages. Tricuspid and pulmonary valve diseases, even in the advanced stages of involvement, may remain asymptomatic for a long time, which can be explained by the low pressure of the pulmonary circulation. Approximately, 57% of patients with moderate‐to‐severe tricuspid insufficiency are either asymptomatic or mildly symptomatic,^2^, ^3^, ^12^ which likely postpones the detection of carcinoid heart involvement without echocardiographic screening. High clinical suspicion and regular follow‐ups are, therefore, needed to establish a timely diagnosis.^3^, ^12^ Primary symptoms usually consist of exertional fatigue and dyspnea. Concurrently with tumor progression and increased levels of serotonin, progressive right‐sided heart failure, accompanied by symptom exacerbation, is likely to appear.^12^ The main findings on physical examinations include edema and elevated jugular venous pressure. With severe tricuspid insufficiency, the V‐wave may be prominent in the jugular venous pulse and a palpable RV impulse might be detected. Additionally, murmurs of tricuspid and pulmonic valve regurgitation may be audible, even though auscultatory findings are mostly negligible due to low‐pressure resistance in the right circulation. Another clinical feature might be systolic murmurs of pulmonic stenosis. In rare instances of left‐sided disease, it may be possible to auscultate murmurs of mitral and aortic regurgitation. It is also worthy of special note that patients may show central cyanosis in the presence of right‐to‐left shunts.^4^, ^10^

#### 3.1.1 Imaging

In the primary evaluation of patients with CaHD, the modality of choice is 2D echocardiography in that it can reveal thickness and retraction in the valve leaflets in a semi‐open position with reduced mobility, annular constriction, thickness and fusion in the subvalvular apparatus, and finally regurgitation and /or stenosis of various degrees.^1^, ^10^, ^11^, ^12^, ^13^ Severe tricuspid regurgitation results in RV volume overload and RV/RA dilatation.^8^, ^14^, ^15^ The findings of 2D echocardiography are summarized in Table 1.

**TABLE 1:** Echocardiographic features of carcinoid heart disease^8^, ^9^, ^10^

Tricuspid valve	Most valvular diseases Thickened, retracted leaflets with reduced mobility and subvalvular involvement Mostly presenting with isolated regurgitation; smaller cases presenting with mixed stenosis and regurgitation Different degrees of tricuspid regurgitation “Dagger‐shaped” in the Doppler profile in severe tricuspid regurgitation (an increase in early peak pressure with a subsequent rapid drop)
Pulmonary valve	Thickened, retracted leaflets with reduced mobility Mostly a combination of stenosis and regurgitation Short pressure half‐time with no flow time in the Doppler profile in severe pulmonary insufficiency
Right atrium/right ventricle	Enlargement with dysfunction Right ventricular outflow tract obstruction may occur. Reduced right ventricular strain (regardless of valvular disease, in some cases even reduced global left ventricular strain)
Left heart valve	Mostly regurgitation with mild‐to‐moderate severity
Metastasis	Direct metastasis into the myocardium as a well‐defined mass

In a review of the echocardiographic findings of 74 patients with CaHD, 100% of the study patients exhibited tricuspid regurgitation, 81% pulmonary regurgitation, 53% pulmonic stenosis, and 7% left‐sided involvement mostly due to the presence of patent foramen ovale (87%) or primary lung neuroendocrine neoplasia (13%).

**FIGURE 2:**
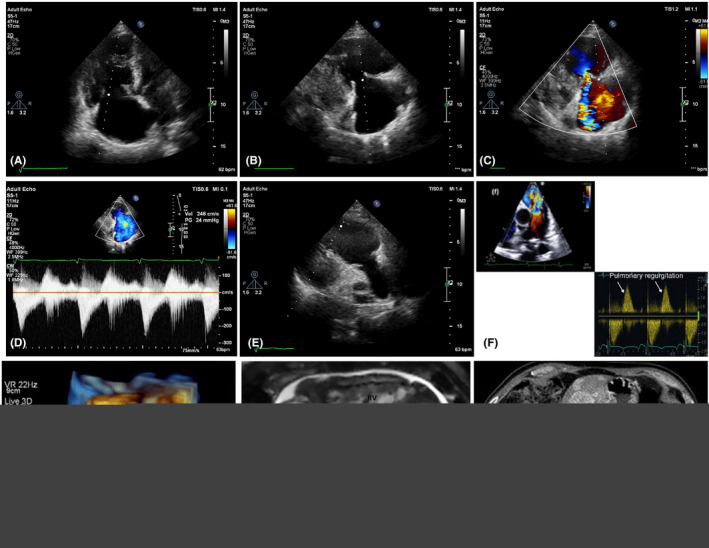
Carcinoid heart disease. A, TTE in the 4‐chamber view shows RV and RA enlargement with thickened, retracted anterior and septal TV leaflets, leading to the malcoaptation of the TV leaflets. B, RV‐inflow tract view in TTE shows thickened and shortened anterior and posterior leaflets on TV. C, Color Doppler study shows severe free tricuspid regurgitation. D, Doppler profiles shows classical “dagger‐shaped” and low‐pressure TR. E, Parasternal short‐axis view shows thickened, retracted TV leaflets. F, Doppler profile shows severe PI in color Doppler study with short PHT of PI. G, Three‐dimensional TEE shows thickened and malcoapted TV leaflets with a fixed systolic defect in systole. H, Cardiac MRI shows carcinoid involvement of TV leaflets. I, Multiple liver metastases of gastrointestinal tumors are visualized herein. TTE, Transthoracic echocardiography; RV, Right ventricle; RA, Right atrium; TV, Tricuspid valve; TR, Tricuspid regurgitation; PV, Pulmonary valve, PI, Pulmonary insufficiency; PHT, Pressure half‐time; MRI, Magnetic resonance imaging

A comparison between 2D TTE and 3D TTE shows that the latter confers some prominent advantages. The 3D modality allows for a detailed assessment of valvular involvement and the surrounding structures and a more accurate evaluation of myocardial masses in cases of direct metastasis.^16^ According to an expert statement by the American College of Cardiology (ACC) concerning the diagnosis and management of CaHD, an echocardiogram should be obtained in all patients with carcinoid syndrome and high suspicion of CaHD. In patients with established CaHD, echocardiography should be repeated every 3‐6 months or during alterations in the clinical status. In contrast, the guidelines of the European Neuroendocrine Tumor Society (ENETS) recommend annual TTE assessments among patients with known CaHD.^2^, ^16^ Although 2D echocardiography offers such distinct advantages as availability, low cost, and lack of radiation, it is not free of limitations and other modalities like cardiac magnetic resonance imaging or computed tomography (CT) could be helpful in these areas. Cardiac magnetic resonance imaging allows for the accurate assessment of chamber size and regurgitation volume, the precise evaluation of valvular dysfunction, and the identification of the infrequent metastases of neuroendocrine neoplasms to the myocardium and the extracardiac structures. Cardiovascular CT findings are deemed exceedingly useful in operative planning inasmuch as they confer an accurate assessment of chamber size, regurgitation volume, and valvular dysfunction severity (Figure 2).^16^ Still, it should be borne in mind that overt CaHD may be diagnosed easily by 2D echocardiography, but the detection of the disease in its early stages might pose a formidable challenge.

The accuracy of echocardiography as a screening tool can be enhanced through the use of sensitive and specific biochemical markers associated with the presence and severity of cardiac involvement. Among these useful biomarkers is N‐terminal pro‐brain natriuretic peptide (NT‐proBNP), which is significantly raised in patients with CaHD. This elevation is correlated with disease progression, symptomatic status, and overall survival. An evaluation of NT‐proBNP with a cutoff level of 260 pg/mL is recommended as a screening biomarker with 92% sensitivity, 91% specificity, 98% negative predictive value, and 71% positive predictive value.

The plasma or urinary level of 5‐hydroxyindoleacetic acid (5‐HIAA), which is a metabolite of serotonin, is higher in cardiac involvement, with an elevated level of this metabolite in excess of 300 mmol/24 h being associated with a two‐ to threefold increased risk of the progression of CaHD.^1^, ^9^

#### 3.1.2 Treatment

The early detection and, thus, timely treatment of CaHD may prevent right heart failure and improve overall survival. The management of these patients is complex and calls for a multidisciplinary approach via a close collaboration between oncologists, cardiologists with expertise in the field of CaHD, and cardiac surgeons. The final goal is to delay the progression of the disease. As the rate of progression is not predictable, regular follow‐ups and intensive monitoring are also required to detect cardiac involvement in the early stages and to determine the timing of surgery before the advanced disease.^9^, ^10^, ^11^ The treatment of cardiac problems in carcinoid syndrome is based not only on the management of volume status and heart failure symptoms but also on the treatment of neuroendocrine neoplasia itself and the reduction in the production of related hormones, as well as ultimately, heart valve surgery.^17^ Volume management is necessary, and diuretics (most commonly loop diuretics with aldosterone antagonists or thiazide, if needed) together with fluid and salt restriction are advised.^4^, ^18^ Nonetheless, caution should be exercised to avoid intravascular depletion in patients with CaHD regardless of the severity of RV dilation or failure because it results in low cardiac output and the manifestation of its symptoms, including light‐headedness, syncope, and fatigue. Other conventional heart failure medications like beta‐blockers, angiotensin‐converting enzyme inhibitors, and angiotensin receptor blockers may be prescribed in some cases, but their efficacy still requires further research.

All of these medications are merely palliative and cannot prevent the progression of the underlying carcinoid syndrome or cardiac involvement.^4^ The pharmacological treatment of CaHD is largely allied to the management of the levels of vasoactive hormones by somatostatin analogs. These medications, including octreotide and lanreotide, are somatostatin receptor inhibitors and decrease the secretion of serotonin and its metabolites (eg, 5‐HIAA), hence their value in the control of symptoms.

For patients who are refractory to somatostatin analogs, the new strategy, incorporating the use of everolimus, interferon‐alpha, peptide‐receptor radionuclide therapy, and telotristat etiprate, has shown great promise.^3^, ^4^, ^9^, ^10^ Another therapeutic option is the prescription of bosentan, which is a dual endothelin receptor antagonist, for the prevention of valvular and mural fibrosis expansion.^10^ The surgical resection of the primary tumors and hepatic metastases seems to decrease the risk of cardiac progression. In the more extensive disease, hepatic intra‐arterial therapies such as embolization can be performed alternatively. Be that as it may, the efficacy of such therapies in stymying the progression of the disease is limited.^9^, ^10^

The effective management of a patient with CaHD is valve surgery in optimal time by an experienced cardiac surgeon aided by a multidisciplinary team with a view to minimizing the adverse effects of the disease and improving the patient's quality of life. What, however, constitutes the appropriate time for the surgical intervention is currently far from clear. There are different scoring systems, principally based on the tricuspid involvement, but the current guidelines generally recommend valve surgery in the presence of severe symptoms or severe valvular disease or progressive asymptomatic RV dysfunction and dilatation.^4^, ^9^, ^10^ Some investigators have advised cardiac surgery in groups of patients who have controlled baseline carcinoid tumors, with at least 12 months of anticipated postoperative survival from their neuroendocrine neoplasia.^8^ Limited evidence is available in favor of surgery in asymptomatic patients. Indeed, the results of the surgical intervention are not conclusive in asymptomatic patients.^9^, ^10^ Møller and colleagues reported a high perioperative survival rate in the early intervention in asymptomatic or mildly symptomatic patients when compared with severely symptomatic patients.^19^

Valve replacement is more common, although extensive fibrotic changes render repair impracticable in most cases. In stenotic lesions not amenable to valve surgery, balloon valvuloplasty is an alternative approach; nevertheless, short‐term hemodynamic and functional benefits and disease relapse diminish the value of this procedure. A controversial topic in the field of valve surgery is the selection of valve prostheses. On the one hand, there is concern regarding bioprosthetic valve degeneration^10^, ^12^ even as early as 3 months after implantation because of the intractably high levels of the vasoactive products that induce carcinoid plaque reformation.^1^, ^12^ On the other hand, mechanical prosthetic valves need lifelong anticoagulation therapy in patients who are already prone to bleeding due to hepatic dysfunction.^1^, ^8^, ^10^ The decision‐making process for the selection of the appropriate type of valve prosthesis is complex and should be individualized based on patient risk assessment. The advantages and disadvantages in this regard, summarized in Table 2, should be explained in detail to the patient as a part of the decision‐making process. The new generation of prosthetic valves with more durability may offer the hope of a more successful rate of valve surgery with less recurrence. Postoperative management should be aimed at maintaining the control of hormone levels and preventing the recurrence of the disease.^12^ During valve surgery, patent foramen ovale should be closed.^8^

**TABLE 2:** Advantages and disadvantages of valvular prostheses in carcinoid heart disease^11^

Valvular prostheses	Advantages	Disadvantages
Mechanical	No carcinoid involvement	Long‐term coagulation therapy
Bioprosthetic	Better short‐term outcomes Valve durability Uncommon carcinoid involvement Avoidance of long‐term warfarin use and secondary coagulopathies	Carcinoid plaque deposition Prosthetic degeneration Thrombus organization
Bioprosthetic (stentless)	No need for long‐term coagulation	Short durability Potential of restenosis Higher incidence of reintervention
Homograft	No need for long‐term coagulation	Homograft constriction Homograft calcification Plaque deposition Premature dysfunction with accelerated stenosis

## 4 CONCLUSIONS

Carcinoid syndrome presents as a paraneoplastic symptom of rare neuroendocrine neoplasia; however, a well‐recognized complication of this syndrome, cardiac involvement, exerts a significant impact on morbidity and mortality. In patients with CaHD, the involvement of right heart valves is more frequent. The treatment of CaHD requires a multidisciplinary approach. Echocardiographic parameters, in conjunction with other imaging modalities and laboratory tests, can assist in determining the optimal time for surgery.

## CONFLICT OF INTEREST

The Authors have no conflict of interest to declare.

## AUTHORS' CONTRIBUTION

AA, GB, and PM: conceptualized and designed the study. AA, GB: involved in literature review and manuscript writing. All the authors have discussed and interpreted the data and commented on the manuscript.

## INFORMED CONSENT

Written informed consent was obtained from the patient for publication of this manuscript and any accompanying image.

## Data Availability

All data relevant to the study are included in the article or uploaded as supplementary information.
